# Supplementary material for: An open-source framework for large-scale, flexible evaluation of biomedical text mining systems
Source: J Biomed Discov Collab. 2008 Jan 29;3:1. doi: 10.1186/1747-5333-3-1 (PMC2276192; doi:10.1186/1747-5333-3-1)
Supplement: Additional file 1 — Evaluation platform source code. The additional file contains the software evaluation framework discussed in this paper. The most current release can be downloaded from SourceForge.net [1]. See the accompanying README file for instructions on installation and use. Also included in the distribution is a collection of UIMA wrappers for some commonly used BioNLP tools and annotated corpora. [file 1747-5333-3-1-S1.zip › bionlp_uima_repository-1.0.1/docs/api/edu/uchsc/ccp/uima/mention/CCPClassMention.html]

CCPClassMention (Center for Computational Pharmacology Util/UIMA API)


|  |  |  |
| --- | --- | --- |
| **Overview** \| **Package** \| **Class** \| **Tree** \| **Deprecated** \| **Index** \| **Help** | |  |
| PREV CLASS   **NEXT CLASS** | **FRAMES**    **NO FRAMES**     **All Classes** |  |
| SUMMARY: NESTED \| FIELD \| CONSTR \| METHOD | DETAIL: FIELD \| CONSTR \| METHOD |  |


---


## edu.uchsc.ccp.uima.mention Class CCPClassMention

```
java.lang.Object
  org.apache.uima.cas.impl.FeatureStructureImpl
      org.apache.uima.jcas.cas.TOP
          edu.uchsc.ccp.uima.mention.CCPMention
              edu.uchsc.ccp.uima.mention.CCPClassMention
```

**All Implemented Interfaces:**: Cloneable, org.apache.uima.cas.FeatureStructure

---

``` public class CCPClassMention extends CCPMention ```

The CCP ClassMention is the root of a flexible class structure that can be used to store virtually any frame-based representation of a particular class. Common class mention types include, but are not limited to, such things as entities (protein, cell type, cell line, disease, tissue, etc.) and frames (interaction, transport, regulation, etc.).
Updated by JCasGen Tue Aug 28 16:44:10 MDT 2007
XML source: /home/williamb/eclipse/RELEASED\_SOURCE\_CODE/descriptors/typeSystem/CCPTypeSystem.xml

---

| **Field Summary** | |
| --- | --- |
| `static int` | `type` |
| `static int` | `typeIndexID` |

**Fields inherited from class org.apache.uima.jcas.cas.TOP**

`addr, jcasType`


| **Constructor Summary** | |
| --- | --- |
| `protected` | `CCPClassMention()`             Never called. |
|  | `CCPClassMention(int addr, org.apache.uima.jcas.cas.TOP_Type type)`             Internal - constructor used by generator |
|  | `CCPClassMention(org.apache.uima.jcas.JCas jcas)` |


| **Method Summary** | |
| --- | --- |
| `org.apache.uima.jcas.cas.FSArray` | `getCcpTextAnnotations()`             getter for ccpTextAnnotations - gets Just as CCPTextAnnotations are linked to a CCPClassMention, it is sometimes useful to be able to follow a CCPClassMention back to its corresponding CCPTextAnnotations, therefore, this FSArray contains links to the CCPTextAnotation(s) for this class. |
| `CCPTextAnnotation` | `getCcpTextAnnotations(int i)`             indexed getter for ccpTextAnnotations - gets an indexed value - Just as CCPTextAnnotations are linked to a CCPClassMention, it is sometimes useful to be able to follow a CCPClassMention back to its corresponding CCPTextAnnotations, therefore, this FSArray contains links to the CCPTextAnotation(s) for this class. |
| `org.apache.uima.jcas.cas.FSArray` | `getSlotMentions()`             getter for slotMentions - gets A class mention optionally has slot mentions which represent attributes of that class. |
| `CCPSlotMention` | `getSlotMentions(int i)`             indexed getter for slotMentions - gets an indexed value - A class mention optionally has slot mentions which represent attributes of that class. |
| `int` | `getTypeIndexID()` |
| `void` | `setCcpTextAnnotations(org.apache.uima.jcas.cas.FSArray v)`             setter for ccpTextAnnotations - sets Just as CCPTextAnnotations are linked to a CCPClassMention, it is sometimes useful to be able to follow a CCPClassMention back to its corresponding CCPTextAnnotations, therefore, this FSArray contains links to the CCPTextAnotation(s) for this class. |
| `void` | `setCcpTextAnnotations(int i, CCPTextAnnotation v)`             indexed setter for ccpTextAnnotations - sets an indexed value - Just as CCPTextAnnotations are linked to a CCPClassMention, it is sometimes useful to be able to follow a CCPClassMention back to its corresponding CCPTextAnnotations, therefore, this FSArray contains links to the CCPTextAnotation(s) for this class. |
| `void` | `setSlotMentions(org.apache.uima.jcas.cas.FSArray v)`             setter for slotMentions - sets A class mention optionally has slot mentions which represent attributes of that class. |
| `void` | `setSlotMentions(int i, CCPSlotMention v)`             indexed setter for slotMentions - sets an indexed value - A class mention optionally has slot mentions which represent attributes of that class. |

**Methods inherited from class edu.uchsc.ccp.uima.mention.CCPMention**

`getMentionName, setMentionName`

**Methods inherited from class org.apache.uima.jcas.cas.TOP**

`addToIndexes, addToIndexes, equals, getAddress, getCAS, getCASImpl, getLowLevelCas, hashCode, removeFromIndexes, removeFromIndexes`

**Methods inherited from class org.apache.uima.cas.impl.FeatureStructureImpl**

`clone, getBooleanValue, getByteValue, getDoubleValue, getFeatureValue, getFeatureValueAsString, getFloatValue, getIntValue, getLongValue, getShortValue, getStringValue, getType, prettyPrint, prettyPrint, prettyPrint, setBooleanValue, setByteValue, setDoubleValue, setFeatureValue, setFeatureValueFromString, setFloatValue, setIntValue, setLongValue, setShortValue, setStringValue, toString, toString`

**Methods inherited from class java.lang.Object**

`finalize, getClass, notify, notifyAll, wait, wait, wait`

**Field Detail**

### typeIndexID

```
public static final int typeIndexID
```

---


### type

```
public static final int type
```


**Constructor Detail**

### CCPClassMention

```
protected CCPClassMention()
```

:   Never called. Disable default constructor

---


### CCPClassMention

```
public CCPClassMention(int addr,
                       org.apache.uima.jcas.cas.TOP_Type type)
```

:   Internal - constructor used by generator

---


### CCPClassMention

```
public CCPClassMention(org.apache.uima.jcas.JCas jcas)
```


**Method Detail**

### getTypeIndexID

```
public int getTypeIndexID()
```

:   **Overrides:**: `getTypeIndexID` in class `CCPMention`

---


### getSlotMentions

```
public org.apache.uima.jcas.cas.FSArray getSlotMentions()
```

:   getter for slotMentions - gets A class mention optionally has slot mentions which represent attributes of that class. These slot mentions are stored in the slotMentions FSArray. There are two types of slot mentions, complex and non-complex. The difference between complex and non-complex slot mentions is simply the type of filler (or slot value) for each. Complex slot mentions are filled with a class mention, whereas non-complex slot mentions are filled by simple Strings.

---


### setSlotMentions

```
public void setSlotMentions(org.apache.uima.jcas.cas.FSArray v)
```

:   setter for slotMentions - sets A class mention optionally has slot mentions which represent attributes of that class. These slot mentions are stored in the slotMentions FSArray. There are two types of slot mentions, complex and non-complex. The difference between complex and non-complex slot mentions is simply the type of filler (or slot value) for each. Complex slot mentions are filled with a class mention, whereas non-complex slot mentions are filled by simple Strings.

---


### getSlotMentions

```
public CCPSlotMention getSlotMentions(int i)
```

:   indexed getter for slotMentions - gets an indexed value - A class mention optionally has slot mentions which represent attributes of that class. These slot mentions are stored in the slotMentions FSArray. There are two types of slot mentions, complex and non-complex. The difference between complex and non-complex slot mentions is simply the type of filler (or slot value) for each. Complex slot mentions are filled with a class mention, whereas non-complex slot mentions are filled by simple Strings.

---


### setSlotMentions

```
public void setSlotMentions(int i,
                            CCPSlotMention v)
```

:   indexed setter for slotMentions - sets an indexed value - A class mention optionally has slot mentions which represent attributes of that class. These slot mentions are stored in the slotMentions FSArray. There are two types of slot mentions, complex and non-complex. The difference between complex and non-complex slot mentions is simply the type of filler (or slot value) for each. Complex slot mentions are filled with a class mention, whereas non-complex slot mentions are filled by simple Strings.

---


### getCcpTextAnnotations

```
public org.apache.uima.jcas.cas.FSArray getCcpTextAnnotations()
```

:   getter for ccpTextAnnotations - gets Just as CCPTextAnnotations are linked to a CCPClassMention, it is sometimes useful to be able to follow a CCPClassMention back to its corresponding CCPTextAnnotations, therefore, this FSArray contains links to the CCPTextAnotation(s) for this class. NOTE: The use of an FSArray is probably not needed here as there is typically only one occupant in the FSArray.

---


### setCcpTextAnnotations

```
public void setCcpTextAnnotations(org.apache.uima.jcas.cas.FSArray v)
```

:   setter for ccpTextAnnotations - sets Just as CCPTextAnnotations are linked to a CCPClassMention, it is sometimes useful to be able to follow a CCPClassMention back to its corresponding CCPTextAnnotations, therefore, this FSArray contains links to the CCPTextAnotation(s) for this class. NOTE: The use of an FSArray is probably not needed here as there is typically only one occupant in the FSArray.

---


### getCcpTextAnnotations

```
public CCPTextAnnotation getCcpTextAnnotations(int i)
```

:   indexed getter for ccpTextAnnotations - gets an indexed value - Just as CCPTextAnnotations are linked to a CCPClassMention, it is sometimes useful to be able to follow a CCPClassMention back to its corresponding CCPTextAnnotations, therefore, this FSArray contains links to the CCPTextAnotation(s) for this class. NOTE: The use of an FSArray is probably not needed here as there is typically only one occupant in the FSArray.

---


### setCcpTextAnnotations

```
public void setCcpTextAnnotations(int i,
                                  CCPTextAnnotation v)
```

:   indexed setter for ccpTextAnnotations - sets an indexed value - Just as CCPTextAnnotations are linked to a CCPClassMention, it is sometimes useful to be able to follow a CCPClassMention back to its corresponding CCPTextAnnotations, therefore, this FSArray contains links to the CCPTextAnotation(s) for this class. NOTE: The use of an FSArray is probably not needed here as there is typically only one occupant in the FSArray.


---


|  |  |  |
| --- | --- | --- |
| **Overview** \| **Package** \| **Class** \| **Tree** \| **Deprecated** \| **Index** \| **Help** | |  |
| PREV CLASS   **NEXT CLASS** | **FRAMES**    **NO FRAMES**     **All Classes** |  |
| SUMMARY: NESTED \| FIELD \| CONSTR \| METHOD | DETAIL: FIELD \| CONSTR \| METHOD |  |


---
